# Dietary modulation of the gut resistome: ecological and metabolic pathways driving antimicrobial resistance

**DOI:** 10.3389/fnut.2026.1868638

**Published:** 2026-06-22

**Authors:** Kumar D. Gahlot

**Affiliations:** 1 Department of Molecular Biology, Umeå University, Umeå, Sweden; 2 Umeå Centre for Microbial Research (UCMR), Umeå, Sweden

**Keywords:** antibiotic tolerance, antimicrobial resistance, dietary modulation, gut microbiome, horizontal gene transfer, host–microbe interactions, microbial ecology, microbiota-accessible carbohydrates

## Abstract

Antimicrobial resistance (AMR) is traditionally viewed as a consequence of antibiotic exposure and genetic adaptation; however, resistance also emerges from the ecological and metabolic context of microbial communities. The human gut microbiome represents a major reservoir of antibiotic resistance genes (ARGs), and diet is increasingly recognised as a dominant regulator of its structure and function. Here, I synthesise current evidence and propose a conceptual framework in which diet shapes resistome dynamics through three interrelated pathways: ecological selection, metabolic regulation, and physicochemical modulation of horizontal gene transfer. Dietary components influence microbial composition, metabolic activity, and the spatial organisation of fermentation along the colon. Diverse fibre types differentially regulate short-chain fatty acid production and microbial competition, whereas high-fat, low-diversity diets destabilise communities and favour opportunistic taxa. Beyond macronutrients, food additives and the physical structure of food alter gut barrier function, microbial stress responses, and spatial ecology, thereby influencing resistome stability. Diet-induced metabolic states further determine antibiotic susceptibility, including transitions between tolerance and resistance. Taken together, this integrated ecological perspective positions diet as a modifiable driver of AMR and highlights nutritional strategies as complementary approaches to mitigating resistome expansion.

## Diet as an ecological driver of the gut resistome

The gut microbiome constitutes a major reservoir of antibiotic resistance genes (ARGs), reflecting long-term ecological selection pressures rather than solely recent antibiotic exposure (1, 2). Increasingly, antimicrobial resistance (AMR) is understood as an emergent property of microbial ecosystems shaped by environmental inputs, among which diet represents one of the most consistent and dominant drivers (3). By continuously modulating resource availability, diet influences microbial composition, diversity, and interspecies competition, thereby structuring the ecological landscape in which ARGs persist and propagate.

Microbiota-accessible carbohydrates (MACs)—including resistant starches, inulin-type fructans, and plant-derived polysaccharides—play a central role in this ecological regulation. These fibre classes differ substantially in fermentability and microbial accessibility, resulting in distinct metabolic outputs and niche differentiation within the gut microbiome (4, 5). A key outcome of MACs fermentation is the production of short-chain fatty acids (SCFAs), which exert diverse ecological and physiological effects. SCFAs, butyrate supports epithelial barrier integrity and anti-inflammatory signalling, thereby limiting pathogen expansion, whereas acetate and propionate contribute to luminal acidification and metabolic cross-feeding networks that stabilise microbial communities (6, 7). Consequently, the impact of dietary fibre on the resistome depends not only on its abundance but also on its compositional diversity and fermentation profile.

Dietary patterns strongly shape these ecological dynamics. High-fibre diets promote microbial diversity and reinforce anaerobic community structure, enhancing colonisation resistance and suppressing opportunistic, ARG-carrying taxa (8, 9). In contrast, high-fat, low-diversity diets disrupt these equilibria by altering bile acid composition and intestinal redox conditions, favouring facultative anaerobes such as *Escherichia coli* and *Enterococcus faecium*, which frequently harbour mobile ARGs (10). Such ecological simplification reduces competitive exclusion and creates niches that facilitate persistence and expansion of resistant populations.

Dietary effects are particularly pronounced following antibiotic perturbation, when microbial ecosystems are destabilised and ecological niches become available. Under these conditions, fibre-rich diets can accelerate microbiome recovery, restore metabolic activity, and re-establish competitive exclusion, whereas nutrient-poor diets prolong dysbiosis and increase resistome instability (11, 12).

**Figure 1:**
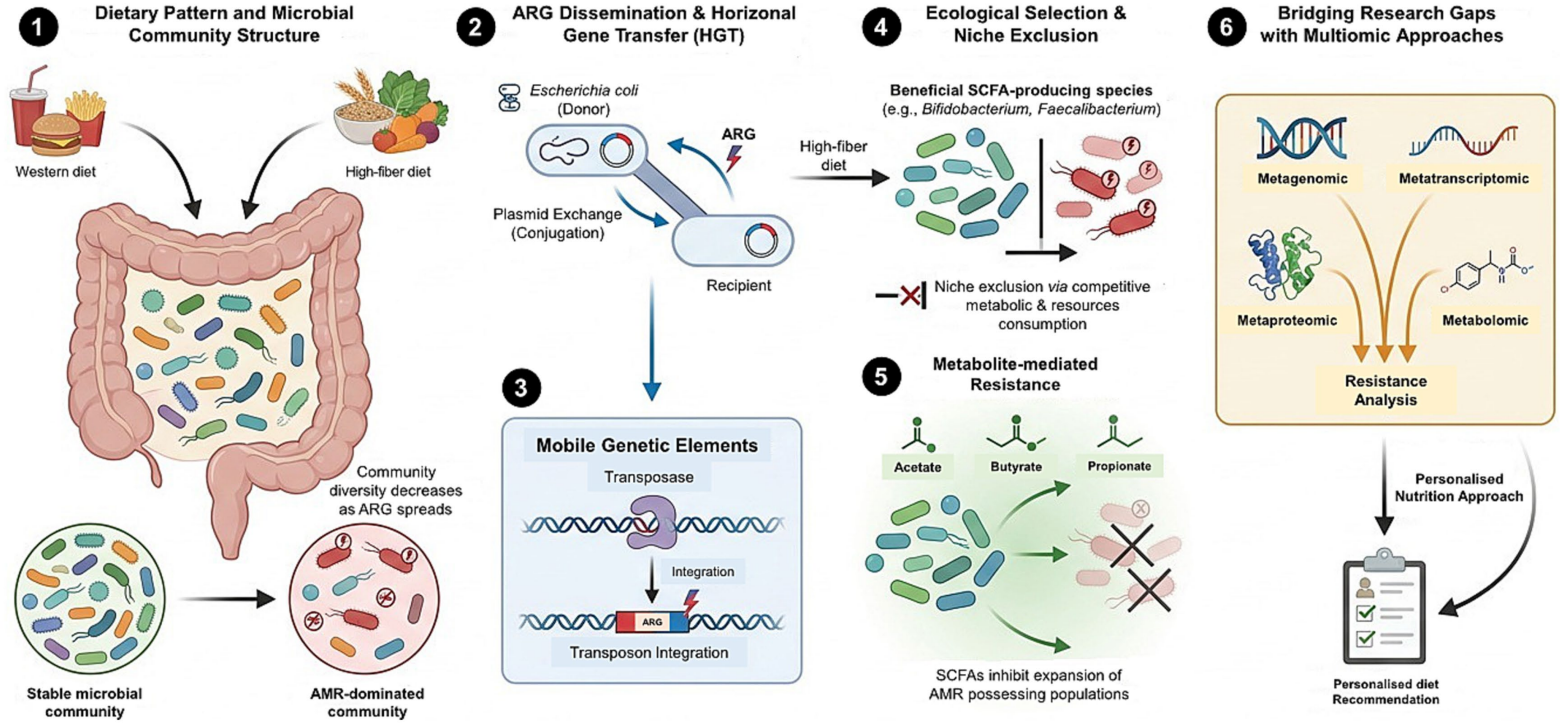
Dietary modulation of the gut resistome through ecological, metabolic, and gene-transfer mechanisms. Dietary inputs regulate antimicrobial resistance *via* interconnected pathways. Fiber-rich diets promote microbial diversity, short-chain fatty acid (SCFA) production, and anaerobic conditions that enhance colonisation resistance, suppress opportunistic taxa, and reduce horizontal gene transfer (HGT). In contrast, high-fat, low-diversity diets disrupt microbial stability, alter bile acid metabolism, and promote expansion of facultative pathogens such as *Escherichia coli* and *Enterococcus faecium*, facilitating antibiotic resistance genes (ARGs) enrichment. ARG dissemination occurs through conjugation, transformation, and phage-mediated transduction, processes that are modulated by environmental stress, microbial density, and metabolic state. Metabolites such as SCFAs contribute to niche exclusion by lowering luminal pH, inhibiting pathogen growth, and enhancing epithelial barrier function. These ecological and metabolic feedbacks collectively determine resistome stability. Integration of multi-omics approaches—including metagenomics, metatranscriptomics, metabolomics, and proteomics—enables high-resolution characterisation of resistome dynamics and supports the development of precision nutrition strategies to mitigate antimicrobial resistance.

Together, these findings position diet as a central ecological determinant of AMR, linking nutrient inputs to microbial community structure, metabolic function, and the long-term dynamics of the gut resistome (Figure 1).

## Physical structure of diet and spatial ecology

The ecological influence of diet extends beyond nutrient composition to include the structural and physicochemical properties of food. The food matrix governs how nutrients are released, fermented, and spatially distributed along the gastrointestinal tract, thereby shaping microbial activity and ecological interactions. This spatial organisation is particularly important in the colon, where gradients in substrate availability, pH, redox state, and microbial density generate functionally distinct niches that support diverse microbial processes (13, 14).

Rapidly fermentable carbohydrates are primarily metabolised in the proximal colon, whereas slowly fermentable substrates, such as resistant starch and complex plant fibres, persist into distal regions (5). This delayed fermentation plays a critical ecological role, as the distal colon is more susceptible to proteolytic metabolism, increased pH, and opportunistic bacterial colonisation. Sustained SCFA production in these distal regions lowers luminal pH, limits pathogen expansion, and reinforces colonisation resistance, thereby constraining the persistence of ARG-harbouring taxa (6, 7).

The physical properties of dietary fibres further influence microbial behaviour and spatial organisation. Viscous fibres increase luminal viscosity, which can restrict the diffusion of metabolites, signalling molecules, and mobile genetic elements, thereby altering microbial interaction networks and gene exchange potential (4). In addition, particle size and encapsulation of nutrients regulate microbial accessibility and fermentation kinetics, creating spatial heterogeneity in substrate utilisation (15). These structural constraints contribute to micro-scale habitat formation within the gut, influencing microbial cooperation, competition, and the likelihood of horizontal gene transfer (HGT) events.

Altogether, the food matrix represents an additional layer of ecological regulation linking diet to resistome dynamics. By shaping the spatial and physicochemical environment of the gut, structural properties of food influence not only microbial composition and metabolism but also the stability and dissemination of AMR, highlighting that diet quality must be understood in both compositional and physical terms.

## Dietary modulation of antibiotic tolerance and resistance

Antibiotic tolerance and resistance represent distinct but interconnected biological states that are both strongly influenced by the dietary environment. Tolerance refers to a reversible physiological condition in which bacteria survive transient antibiotic exposure without acquiring genetic changes, whereas resistance involves stable genetic adaptations that enable growth in the presence of antibiotics. Importantly, tolerance is increasingly recognised as a precursor to resistance evolution, as it extends bacterial survival and provides a temporal window for mutation or acquisition of ARGs (16, 17).

Diet directly modulates tolerance by shaping microbial metabolism and physiological states. Nutrient availability regulates bacterial growth rate, energy production, and redox balance, all of which influence antibiotic susceptibility. Many bactericidal antibiotics rely on active cellular processes, such that reduced metabolic activity diminishes drug efficacy. Experimental studies in the gut microbiome demonstrate that metabolic state is a major determinant of antibiotic effectiveness, linking nutrient conditions directly to treatment outcomes (12, 18). In particular, perturbations in central carbon metabolism and respiratory activity can alter intracellular ATP levels, proton motive force, and antibiotic uptake, thereby modulating tolerance phenotypes.

Dietary patterns can either promote or suppress these tolerance states. Diets high in fat or low in fermentable substrates are associated with altered energy flux, increased oxidative stress, and activation of stress-response pathways, all of which can induce tolerance-like physiological states. Such conditions reduce antibiotic efficacy by promoting persistence and slowing growth. Indeed, dietary composition itself has been shown to act as a source of antibiotic tolerance within microbial communities (19). These tolerant populations are of particular concern because they survive treatment and provide a reservoir for subsequent resistance evolution.

Conversely, fibre-rich diets that support fermentation and SCFAs production promote sustained microbial activity and ecological competition. These conditions reduce the prevalence of dormant or persister subpopulations and facilitate recovery of microbiome structure following antibiotic exposure. SCFAs also modulate intracellular pH and metabolic flux, further influencing antibiotic susceptibility and microbial fitness (6).

Collectively, these findings highlight that diet influences not only the emergence of genetic resistance but also the underlying physiological states that enable it. By regulating microbial metabolism and tolerance dynamics, dietary inputs link ecological conditions to evolutionary trajectories of AMR.

## Food additives and gut microbial stress responses

Modern diets include a wide range of additives that influence gut microbial ecology beyond macronutrients composition. Compounds such as emulsifiers, artificial sweeteners, and preservatives can perturb host–microbe interactions by disrupting epithelial barrier integrity, altering microbial composition, and inducing stress responses within microbial communities (20, 21). These effects extend the role of diet from a nutritional input to a regulator of microbial physiology and ecological stability.

Dietary additives have been shown to reduce beneficial commensal populations while promoting inflammatory conditions that favour opportunistic taxa. For example, emulsifier-induced thinning of the mucus layer increases microbial proximity to the epithelium, enhancing immune activation and reshaping community structure toward inflammation-associated microbiota (20). Such shifts can favour taxa with increased adaptive capacity, including those more likely to harbour or acquire ARGs. These changes weaken colonisation resistance and create ecological niches that support persistence of ARG-carrying organisms.

At a mechanistic level, additives can induce microbial stress responses, including oxidative stress, envelope stress, and DNA damage pathways. These stress conditions are closely linked to HGT, as activation of the bacterial SOS response and related regulatory systems can stimulate the mobilisation of genetic elements such as plasmids, integrons, and bacteriophages (22, 23). Increased oxidative and inflammatory stress also enhances microbial turnover and DNA release, further promoting transformation and transduction processes. Thus, additives may indirectly facilitate ARG dissemination by creating physiological conditions that favour gene exchange.

Emerging systems-level studies emphasise that diet–microbiome interactions, including processed food components, play a central role in shaping AMR dynamics (11). These findings highlight that dietary quality and food processing are critical, yet often overlooked, determinants of resistome structure and stability. Incorporating food additives into AMR research expands the ecological framework of resistance, recognising that non-nutrient dietary components can modulate microbial stress responses and gene transfer potential in ways that influence long-term resistance outcomes.

## Fermented foods and probiotics: opportunities and risks

Fermented foods and probiotic interventions offer promising strategies to enhance gut microbial diversity, resilience, and functional stability. These approaches introduce live microorganisms and bioactive metabolites that can reinforce colonisation resistance, modulate host immune responses, and support microbiome recovery following perturbations such as antibiotic treatment (24, 25). From an ecological perspective, such interventions can restore network complexity and functional redundancy, thereby limiting ecological niches available to opportunistic, ARG-harbouring taxa. Additionally, microbial metabolites derived from fermentation, including organic acids and bacteriocins, can directly inhibit pathogen growth and contribute to resistance suppression within the gut ecosystem.

Despite these benefits, the contribution of probiotics to AMR dynamics remains complex. Some probiotic strains carry ARGs, necessitating careful evaluation of their safety profiles. A key distinction lies between transient carriage and stable integration of ARGs. Many probiotics exhibit limited colonisation potential and pass through the gastrointestinal tract without establishing long-term residency, thereby reducing their direct contribution to the resident resistome (26).

However, risk arises when ARGs are associated with mobile genetic elements such as plasmids, integrons, or transposons. Under favourable ecological conditions—including high microbial density, antibiotic exposure, or stress-induced activation of gene transfer pathways—these elements can facilitate HGT to resident commensal or pathogenic bacteria (27, 28). Such transfer events may result in stable incorporation of ARGs into the indigenous microbiota, contributing to long-term resistome expansion (29).

Ecological compatibility between probiotic strains and the resident microbiota further modulates this risk. Strains that successfully integrate into microbial networks may have increased opportunities for gene exchange, whereas transient strains may exert primarily short-term functional effects. Consequently, safety assessment should extend beyond simple ARG detection to include genomic context, transferability, and ecological behaviour.

Overall, fermented foods and probiotics represent valuable tools for microbiome modulation and antimicrobial stewardship (24). However, their application requires a balanced and evidence-based approach that considers both their therapeutic potential and the risk of unintended ARG dissemination within complex gut ecosystems.

## Metabolic and regulatory mechanisms

Diet shapes AMR by modulating bacterial metabolism and regulatory networks that govern physiological responses to environmental cues. Nutrient availability acts as a primary signal influencing gene expression, stress-response pathways, and cellular states that ultimately determine antibiotic susceptibility. Through these mechanisms, diet links environmental inputs to both microbial function and resistance phenotypes (11, 30).

Experimental evidence demonstrates that microbial metabolism directly governs antibiotic effectiveness within the gut microbiome. Changes in carbon source availability and metabolic flux influence intracellular ATP levels, redox balance, and proton motive force, all of which modulate antibiotic uptake and activity (12, 18). Growth rate is a key determinant of susceptibility, as many bactericidal antibiotics target actively dividing cells; thus, reduced metabolic activity can diminish efficacy, whereas metabolically active cells are more vulnerable. These findings highlight that antibiotic susceptibility is not static but dynamically shaped by metabolic state.

At the cellular level, nutrient-responsive regulatory networks integrate metabolic signals with stress responses. Two-component systems and global regulators coordinate adaptation to environmental stressors, including antibiotic exposure. Among these, envelope stress responses play a critical role in modulating membrane composition, permeability, and cell-surface characteristics, thereby directly affecting antibiotic interactions. For example, in *Yersinia pseudotuberculosis*, nutrient-dependent activation of the Cpx stress-response pathway alters cell envelope remodelling and the expression of virulence traits, thereby influencing susceptibility to last-resort antimicrobials (31–33). Importantly, such regulatory mechanisms are conserved across diverse bacterial taxa and extend beyond pathogens to commensal gut microbes.

At the community level, diet-driven metabolic interactions further shape resistance dynamics through cross-feeding and metabolite exchange. SCFAs generated from microbial fermentation of dietary fibers act not only as energy sources but also as signalling molecules that influence microbial gene expression and stress responses (6). These metabolites can modulate intracellular pH, regulate transcriptional programmes, and alter competitive interactions, thereby indirectly influencing resistance phenotypes.

Emerging nutrigenomic studies provide additional insight into how diet regulates resistance-associated gene networks by integrating environmental signals with microbial regulatory systems (30). These findings emphasise that antimicrobial resistance is not solely determined by genetic determinants but is profoundly influenced by metabolic and regulatory contexts shaped by diet.

Collectively, metabolic and regulatory processes provide a mechanistic bridge linking dietary inputs to resistome dynamics, reinforcing the concept that antibiotic susceptibility is a context-dependent phenotype governed by environmental conditions.

## Dietary modulation of horizontal gene transfer

HGT is a central mechanism underlying the dissemination of ARGs within the gut microbiome, enabling rapid adaptation and evolution of microbial communities. While HGT is inherently a genetic process (34), its frequency and efficiency are strongly shaped by ecological and environmental factors, many of which are directly influenced by diet (2, 11).

Dietary modulation of microbial metabolism plays a key role in regulating HGT dynamics. Fermentation of microbiota-accessible carbohydrates produces SCFAs, which lower luminal pH and create anaerobic conditions that can suppress plasmid stability and conjugation efficiency in certain bacterial systems (6, 28). In addition to these direct physicochemical effects, SCFAs enhance epithelial barrier function and reduce inflammation, thereby limiting ecological niches for opportunistic pathogens that often act as reservoirs and donors of ARGs.

Redox conditions further influence gene transfer processes. Diet-induced inflammation, frequently associated with Western dietary patterns, can increase oxygen availability in the gut lumen, favouring facultative anaerobes such as *E. coli* that are efficient mediators of conjugative plasmid transfer (10, 35). Elevated oxidative stress also promotes bacteriophage induction and DNA damage responses, both of which can enhance transduction and transformation pathways, contributing to ARG dissemination (22).

Physical and structural properties of the gut environment represent an additional layer of regulation. Fibre-rich diets increase luminal viscosity and modify spatial organisation, which can restrict the diffusion of plasmids, bacteriophages, and extracellular DNA, thereby limiting opportunities for gene exchange. Conversely, structured microbial communities such as biofilms provide localised environments of high cell density and close proximity, facilitating conjugation and enhancing HGT efficiency (34, 36). These opposing effects underscore the complexity of diet-driven HGT regulation, where the same dietary factor may both constrain and promote gene transfer depending on ecological context.

At the community level, microbial diversity and interaction networks further modulate HGT. Highly diverse ecosystems may reduce horizontal gene exchange through competitive exclusion and niche stability, whereas disrupted or simplified communities often exhibit increased gene flow due to reduced ecological constraints.

Collectively, these findings demonstrate that HGT is not solely determined by genetic elements but is dynamically regulated by diet-driven ecological, metabolic, and physicochemical conditions. This reinforces the concept that dietary interventions can influence not only microbial composition but also the evolutionary trajectories of antimicrobial resistance within the gut ecosystem.

## Conceptual framework

Diet shapes the gut resistome through three interconnected processes: ecological selection, metabolic regulation, and physicochemical modulation of HGT. This framework integrates principles from microbial ecology, systems biology, and evolution to explain how environmental inputs are translated into resistance outcomes across multiple biological scales (2, 11).

At the ecological level, diet determines microbial community composition by shaping resource availability and competitive interactions. Diverse, fibre-rich diets promote species richness, functional redundancy, and colonisation resistance, thereby limiting the expansion of opportunistic, ARG-carrying taxa (3, 4). In contrast, simplified or Western-style diets reduce ecological complexity, creating unstable niches that facilitate invasion and persistence of resistant populations.

At the metabolic level, nutrient availability governs bacterial physiology, including growth rate, energy production, and stress responses. These metabolic states directly influence antibiotic susceptibility and the emergence of tolerance, thereby shaping the likelihood of resistance evolution (12, 18). Importantly, metabolic regulation operates not only at the level of individual cells but also across microbial communities through cross-feeding interactions and shared metabolite pools, linking diet to collective behaviour and community-level resilience.

At the physicochemical level, diet modifies environmental parameters such as pH, redox state, viscosity, and spatial organisation within the gut. These factors regulate the efficiency of HGT by influencing plasmid stability, microbial contact rates, and activation of mobile genetic elements (28, 35). Thus, diet shapes not only which microbes are present and how they function, but also how genetic information is exchanged within the community.

These three processes are deeply interdependent and give rise to dynamic feedback loops. For example, ecological shifts alter metabolic outputs, which in turn modify physicochemical conditions that regulate gene transfer. Conversely, HGT-driven acquisition of new traits can reshape ecological fitness and metabolic capacity, further influencing community structure.

This systems-level framework positions diet as a central regulator of resistome dynamics, linking environmental inputs to microbial ecology, physiology, and evolution. By integrating ecological selection, metabolic regulation, and gene transfer processes, it provides a unifying model for understanding how dietary interventions can influence antimicrobial resistance in complex microbial ecosystems.

## Challenges, limitations, and future perspectives

Inter-individual variability remains a major barrier to translating diet–microbiome–resistome interactions into clinical applications. Differences in host genetics, baseline microbiome composition, lifestyle, and dietary adherence contribute to substantial heterogeneity in microbiome responses, complicating the prediction of dietary effects on AMR outcomes (26, 37). This variability is further compounded by the dynamic nature of the gut microbiome, which responds rapidly to environmental changes yet exhibits individual-specific resilience and stability patterns (10, 38).

Establishing causal relationships between dietary factors and resistance dynamics remains a central challenge. Much of the current evidence is derived from observational studies or short-term interventions, which limits mechanistic interpretation. Controlled dietary trials integrating longitudinal sampling are required to disentangle cause–effect relationships and to identify reproducible signatures of diet-driven resistome modulation. The integration of multi-omics approaches—including metagenomics, metatranscriptomics, metabolomics, and proteomics—provides a powerful framework for linking microbial composition, functional activity, and resistance gene dynamics at high resolution (39).

Another limitation lies in the complexity of dietary exposures. Current frameworks often focus on macronutrient composition, yet emerging evidence suggests that fibre diversity, food matrix structure, and food additives exert independent and interacting effects on microbial ecology and resistance mechanisms. Incorporating these factors into study design is essential to capture the full spectrum of diet-driven influences on the resistome. In particular, the spatial organisation of microbial activity along the gastrointestinal tract remains underexplored, despite its importance in shaping microbial interactions and HGT dynamics (14).

Future research should also prioritise ecological and systems-level modelling approaches to integrate these diverse dimensions. Linking dietary inputs to microbial metabolism, environmental conditions, and gene transfer processes will be critical for developing predictive models of resistance dynamics. Such models could enable personalised dietary interventions aimed at reducing resistome expansion and improving treatment outcomes.

Collectively, addressing these challenges will require interdisciplinary efforts combining nutrition science, microbiology, systems biology, and clinical research. Advancing beyond descriptive associations toward mechanistic and predictive understanding represents a key step in harnessing diet as a viable strategy for mitigating antimicrobial resistance.

## Conclusion

Diet represents a central and modifiable determinant of AMR, shaping the gut resistome through interconnected ecological, metabolic, and physicochemical processes. By influencing microbial diversity, regulating physiological states such as antibiotic tolerance, and modulating the conditions that govern gene transfer, dietary patterns play a decisive role in how resistance emerges, stabilises, and spreads within microbial communities.

This perspective shifts the paradigm from pathogen-focused interventions toward ecosystem-level management, recognising that resistance is an emergent property of complex, diet-shaped microbial environments. Nutritional strategies that promote microbial diversity, sustain metabolic activity, and constrain opportunities for gene exchange have the potential to reinforce colonisation resistance and limit the expansion of resistant populations.

Integrating dietary considerations into antimicrobial stewardship frameworks offers a promising and complementary approach to existing strategies. By addressing the environmental context in which resistance develops, such approaches may enhance the long-term effectiveness of antimicrobial therapies and contribute to more sustainable management of AMR.

## Data Availability

The original contributions presented in the study are included in the article, further inquiries can be directed to the corresponding author.
